# Analysis of Longitudinal Binomial Data with Positive Association between the Number of Successes and the Number of Failures: An Application to Stock Instability Study

**DOI:** 10.3390/e24101472

**Published:** 2022-10-16

**Authors:** Xiaolei Zhang, Guohua Yan, Renjun Ma, Jiaxiu Li

**Affiliations:** 1 Pan-Asia Business School, Yunnan Normal University, Kunming 650031, China; 2 Department of Mathematics and Statistics, University of New Brunswick, Fredericton, NB E3B 5A3, Canada

**Keywords:** best linear unbiased predictors, logistic regression, nonparametric random effects, overdispersion, random cluster sizes, zero inflation, 62J05, 62J12, 62P05

## Abstract

Numerous methods have been developed for longitudinal binomial data in the literature. These traditional methods are reasonable for longitudinal binomial data with a negative association between the number of successes and the number of failures over time; however, a positive association may occur between the number of successes and the number of failures over time in some behaviour, economic, disease aggregation and toxicological studies as the numbers of trials are often random. In this paper, we propose a joint Poisson mixed modelling approach to longitudinal binomial data with a positive association between longitudinal counts of successes and longitudinal counts of failures. This approach can accommodate both a random and zero number of trials. It can also accommodate overdispersion and zero inflation in the number of successes and the number of failures. An optimal estimation method for our model has been developed using the orthodox best linear unbiased predictors. Our approach not only provides robust inference against misspecified random effects distributions, but also consolidates the subject-specific and population-averaged inferences. The usefulness of our approach is illustrated with an analysis of quarterly bivariate count data of stock daily limit-ups and limit-downs.

## 1. Introduction

There is continuing interest in developing mixed models for longitudinal binomial data [1,2]; however, these methods in the literature generally assume a fixed number of trials, and thus imply a negative association between the number of successes and the number of failures. As the number of trials is fixed, an increase in the number of successes implies a decrease in the number of failures, and vice versa. In practice, however, the number of successes and the number of failures are often positively associated when the number of trials is random. That is, the number of successes and the number of failures can increase or decrease simultaneously. This can be illustrated further with a hypothetical example in Table 1. Clearly the sample correlation is 1 although both probabilities of success and failure are 0.5. This is because the two outcomes can increase together if the totals are not fixed. On the other hand, when the totals are fixed, the increase of one outcome is at the loss of the other; therefore, they are negatively associated. Thus, the inference methods for the case of fixed cluster sizes are no longer valid for the case of varying cluster sizes since the key assumption of negative association has been violated [3].

In the analysis of clustered binary data, the importance of accounting for randomness in the cluster sizes has long been recognized in developmental toxicity studies, disease aggregation and behaviour studies [4,5]; therefore, varying cluster sizes are likely to occur if such data are collected longitudinally in these areas. Hence, mixed models that can accommodate a random number of trials are also needed in the analysis of longitudinal binomial analysis of positively associated numbers of successes and numbers of failures. In addition, traditional approaches to mixed models for longitudinal binomial data usually rely on the specification of particular random effects distributions; therefore, concerns over the validity of the assumed random effects distributions and the robustness of such inferences were raised [6].

Our work is motivated by the daily price limit policy in Chinese stock market. Quarterly bivariate counts of stock daily limit-ups and limit-downs were collected over 49 seasons from the second quarter of 2007 to the second quarter of 2019 for 60 randomly selected stocks. The quarterly counts of stock daily limit-ups and the quarterly counts of stock limit-downs were positively correlated over time within every stock; the Pearson correlation coefficient ranged from 0.11 to 0.92 with an average of 0.54 for these 60 stocks. The mixed models for longitudinal binomial data in the literature imply a negative association between the number of successes and the number of failures and are inappropriate for the analysis of quarterly bivariate counts of stock daily limit-ups and limit-downs for these stocks. These binomial mixed models in the literature also imply that the number of trials is always positive; however, more than 58% of the corresponding number of trials for our quarterly bivariate counts of stock daily limit-ups and limit-downs were exactly zero. The research on the price limit of stock mainly focuses on its impact on stock volatility [7,8,9]. It is generally believed that the price limit policy strengthened the herding effect of the market and made stock prices a self-exciting process. This is indeed one of the characteristics of Chinese stock market, that is, stocks are prone to frequent limit-ups and limit-downs. For market managers, in the process of extreme market fluctuations, the limit-ups and limit-downs bring a lack of liquidity, and the market is prone to systemic risks. For investors, the extreme volatility of stocks will directly affect their investment decisions and investment returns; therefore, the instability of the stocks is of great interest in the analysis of extreme price fluctuations but has not been studied so far.

In this paper, we propose a three-level joint Poisson mixed model for both longitudinal counts of successes and longitudinal counts of failures in the longitudinal binomial data with varying numbers of trials over time. Without loss of generality, we describe the model here in terms of quarterly data of stock daily limit-ups and limit-downs to facilitate understanding. First, we introduce stock-specific random effects shared by both counts of stock daily limit-ups and limit-downs. The higher the stock-specific distribution-free random effects, the higher both quarterly counts of stock daily limit-ups and limit-downs; therefore, the stock-specific random effect characterizes the instability of the corresponding stock. Second, conditioning on stock-specific random effects, we introduce two sequences of autocorrelated distribution-free random effects; one for quarterly counts of stock daily limit-ups, whereas the other is for quarterly counts of stock daily limit-downs. Finally, given both stock-specific and the corresponding autocorrelated random effects, we assume that both quarterly counts of stock daily limit-ups and limit-downs follow Poisson distributions with time-dependent covariates. This joint Poisson mixed model can accommodate randomness and zero in the total counts of quarterly stock daily limit-ups and limit-downs at each time point and imply a positive cross association between the quarterly counts of stock daily limit-ups and the quarterly counts of stock limit-downs. Following Ma and Jørgensen [10] and Ma et al. [5], we develop a model estimation based on the orthodox best linear unbiased predictors (BLUP) of random effects given the data. As our approach does not require the specification of any parametric distribution for random effects, our inference is robust against misspecified random effects distributions. To the best of our knowledge, this is the first time a mixed model is developed for longitudinal binomial data where the number of successes and the number of failures are positively associated over time.

The rest of the paper is organized as follows. After introducing quarterly count data of stock daily limit-ups and limit-downs in Section 2, we propose a joint model for bivariate longitudinal counts and discuss its implied longitudinal binomial model in Section 3. In Section 4, we discuss the orthodox best linear unbiased predictors of random effects and model estimation. We analyze the quarterly stock price limits data in Section 5 and conclude in Section 6.

## 2. Quarterly Data of Stock Daily Limit-Ups and Limit-Downs

A unique daily price limit policy has been in place in Chinese stock market since 13 December 1996. The purpose of the price limit policy is to reduce the volatility of prices by setting limits on how much each stock can rise or fall on a daily basis. The price can move within 10% of the previous day’s closing price, and a quotation that is outside the range will be invalid. These daily limits on rise or fall are called stock daily limit-ups and limit-downs.

**Figure 1:**
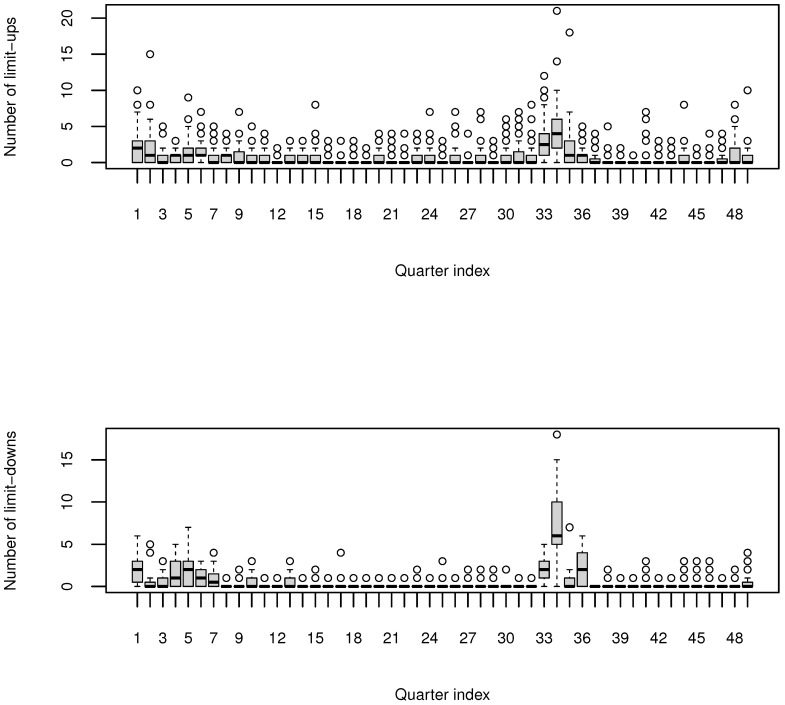
Parallel box plots of quarterly counts of stock daily limit-ups (**top** panel) and limit-downs (**bottom** panel). The quarters are numbered consecutively in the horizontal axis with “1” for the second quarter of 2007 and 49 for the second quarter of 2007.

To characterize the instability of the stocks, quarterly bivariate counts of stock daily limit-ups and limit-downs were collected over 49 seasons from the second quarter of 2007 to the second quarter of 2019 for 60 selected stocks. These 60 stocks were randomly selected from the CSI Small cap 500 index (CSI 500) index in order to make the selected samples generally representative of the market and make the conclusion of this research widely applicable. The data are available from http://www.sse.com.cn/ (accessed on 5 January 2020) and http://www.szse.cn/ (accessed on 5 January 2020). The parallel boxplots of this pair of quarterly counts of stock daily limit-ups and limit-downs are displayed in Figure 1 below. The quarterly counts of stock daily limit-ups range between 0 and 21, whereas the quarterly counts of stock daily limit-downs range between 0 and 18. All these counts are far below their ceiling counts of around 60 (trading days); thus, the ceiling counts are not necessarily a concern for our use of conditional Poisson for the quarterly counts [11].

To study the relationship between quarterly bivariate counts of stock daily limit-ups and limit-downs and basic characteristics of stocks that are prone to rise and fall limits, we also collected information on the following three variables quarterly: price-to-earnings ratio (PE), price-to-book ratio (PB) and price-to-sales ratio (PS). These three variables are common and important growth indicators reflecting stock fundamentals [12,13,14]. Our analysis results are expected to help managers and investors in risk management and investment decision-making.

## 3. Joint Model for Bivariate Longitudinal Counts

### 3.1. The Model

Let $Y_{i1t}$ be the quarterly count of daily limit-ups and $Y_{i2t}$ the quarterly count of daily limit-downs, where *i* indexes one of these 60 stocks, i=1,…,m=60, and t=1,…,T=49 indexes the seasons under study with t=1 corresponding to the second quarter of 2007 and t=49 the second quarter of 2019. Let $N_{it}$ be the total number of limit-ups or limit-downs for the *i*th stock in the *t*th season. We model the quarterly counts of daily limit-ups and limit-downs jointly through a three-level Poisson mixed model as follows.

**Assumption** **1.** *At the top level, we introduce a stock-specific random effect $U_{i}$ for each stock, i=1,…,m. We assume that the $U_{i}$’s are positive, independently and identically distributed with mean 1 and variance $\sigma^{2}$.*

**Assumption** **2.** *At the second level, we introduce two sequences of random effects for quarterly counts of daily limit-ups and limit-downs of each stock, $V_{i1t}$ for the count of daily limit-ups $Y_{i1t}$ and $V_{i2t}$ for the count of daily limit-downs $Y_{i2t}$. Denote $U={\left(U_{1},U_{2},\ldots ,U_{T}\right)}^{\prime }$. Conditioning on U, we assume that these stock × season random effects are positive and identically distributed with*

$$E\left(V_{i1t}|U\right)=E\left(V_{i2t}|U\right)=U_{i}$$

*and*

$$Cov\left(V_{ijt},V_{i^{\prime }j^{\prime }t^{\prime }}|U\right)=\left\{\begin{array}{cc} \tau_{j}^{2}\rho_{j(t,t^{\prime })}U_{i} & if\;i=i^{\prime }\;and\;j=j^{\prime }\\ 0 & if\;i\neq i^{\prime }\;or\;j\neq j^{\prime } \end{array}\right.$$

*with $\rho_{j(t,t^{\prime })}=1$ for $t=t^{\prime }$, $j,j^{\prime }=1,2$. This formulation of correlations of the random effects is general as it encompasses various correlation structures including unstructured, m-dependence, Toeplitz, exchangeable, etc. In this paper, we focus on the first-order autoregression (AR(1)), in which $Cor\left(V_{ijt},V_{ijt^{\prime }}\right)=\rho_{j}^{|t-t^{\prime }|}$.*

**Assumption** **3.** *At the response level, we assume the quarterly counts of limit-ups and limit-downs are conditionally Poisson distributed, given the stock-specific random effects and the stock × season random effects. Denote $V={\left(V_{1}^{\prime },V_{2}^{\prime },\ldots ,V_{m}^{\prime }\right)}^{\prime }$, where $V_{i}=(V_{i1}^{\prime }$, $V_{i2}^{\prime }{)}^{\prime }$, $V_{ij}=(V_{ij1}$, $V_{ij2},\ldots ,V_{ijT}{)}^{\prime }$ and $W={\left(U^{\prime },V^{\prime }\right)}^{\prime }$. Specifically, we assume that counts $Y_{i1t}$’s and $Y_{i2t}$’s are conditionally independent and Poisson distributed with*

(1) $$\left\{\begin{array}{c} Y_{i1t}|W∼Poisson\left(V_{i1t}\exp\left(z_{it}^{\prime }\alpha +x_{it}^{\prime }\beta \right)\right)\\ Y_{i2t}|W∼Poisson\left(V_{i2t}\exp\left(z_{it}^{\prime }\alpha \right)\right) \end{array}\right.$$

*where $x_{it}={\left(x_{it1},x_{it2},\ldots ,x_{itp}\right)}^{\prime }$ and $z_{it}={\left(z_{it1},z_{it2},\ldots ,z_{itp}\right)}^{\prime }$ are known vectors of covariates, **α** and **β** are unknown regression coefficients. Here, we employ the same strategy as in Ma et al. [5] to incorporate covariates in the model. In general, z and x can be different, but they are the same in the analysis of this paper:*

$$z_{it}^{\prime }\alpha =\alpha_{0}+PE\alpha_{1}+PB\alpha_{2}+PS\alpha_{3}\;and\;x_{it}^{\prime }\beta =\beta_{0}+PE\beta_{1}+PB\beta_{2}+PS\beta_{3}.$$

**Remarks**. (1) The proposed joint Poisson mixed model can accommodate both a random and zero number of trials. From Equation (1), given all the random effects, the total number of counts $N_{it}$ is also conditionally independent and Poisson distributed,

(2) $$N_{it}|W∼Poisson\left(V_{i1t}\exp\left(z_{it}^{\prime }\alpha +x_{it}^{\prime }\beta \right)+V_{i2t}\exp\left(z_{it}^{\prime }\alpha \right)\right).$$

Clearly, $N_{it}$ can take a value of zero with positive probability and thus, our model can handle the case that both the quarterly counts of limit-ups and limit-downs are zeros. This is advantageous to traditional logistic mixed models for the number of successes in which the portion of data with zero number of trials has to be excluded from the analysis.

(2) As each of the pair of Poisson mixed models is a generalization of a negative binomial model, our model can accommodate overdispersion and zero inflation.

(3) In Assumptions 1 and 2 above, we only assume the mean and variance structures of the random effects, without specifying any parametric form for their distributions. Furthermore, our estimation method to be discussed in the next section requires only these first two moments of the random effects. Thus, our model is robust against misspecified random effects distributions.

(4) Our model consolidates subject-specific and population-averaged inferences for the number of successes and for the number of failures. Under the model setup, the marginal means of the counts are

(3) $$E\left(Y_{i1t}\right)=\exp\left(z_{it}^{\prime }\alpha +x_{it}^{\prime }\beta \right)\;\mathrm{and}\;E\left(Y_{i2t}\right)=\exp\left(z_{it}^{\prime }\alpha \right)$$

Comparing Equations (1) and (3), it is clear that the regression parameters α and β can be interpreted marginally the same way as conditionally.

(5) The number of successes $Y_{i1t}$ is conditionally binomial, given the number of trials and the random effects:

(4) $$Y_{i1t}|\left(N_{it},W\right)∼binomial\left(N_{it},p_{it}\right),$$

where

$$p_{it}=\frac{V_{i1t}\exp\left(x_{it}^{\prime }\beta \right)}{V_{i1t}\exp\left(x_{it}^{\prime }\beta \right)+V_{i2t}}.$$

Thus, the binomial probability $p_{it}$ is linear in the regression parameters β under the logit link as follows:

(5) $$logit\left(p_{it}\right)=\log\left(\frac{V_{i1t}}{V_{i2t}}\right)+x_{it}^{\prime }\beta .$$

Note that α does not appear in Equation (5); therefore, it is auxiliary in this induced binomial model.

### 3.2. Covariance Structure

We now give the moment structures of the random effects and responses which will be used in the model estimation; these can be obtained after some algebraic calculations by the method of conditioning on random effects. For ease of programming, we present some results in matrix forms.

In addition to the vectors introduced so far, let $Y_{i}^{\prime }$ = ${\left(Y_{i1}^{\prime },Y_{i2}^{\prime }\right)}^{\prime }$ where $Y_{i1}$ = $(Y_{i11}$, $Y_{i12}$, *…*, $Y_{i1T}{)}^{\prime }$ and $Y_{i2}$ = ${\left(Y_{i21},Y_{i22},\ldots ,Y_{i2T}\right)}^{\prime }$. Similarly, let $\mu_{i}$ = ${\left(\mu_{i1}^{\prime },\mu_{i2}^{\prime }\right)}^{\prime }$ where $\mu_{i1}$ = $(\mu_{i11}$, $\mu_{i12}$, *…*, $\mu_{i1T}{)}^{\prime }$, $\mu_{i2}$ = $(\mu_{i21}$, $\mu_{i22}$, *…*, $\mu_{i2T}{)}^{\prime }$, $\mu_{i1t}$ = $\exp(z_{it}^{\prime }\alpha +x_{it}^{\prime }\beta )$ and $\mu_{i2t}$ = $\exp(z_{it}^{\prime }\alpha )$. The means of the random effects and the responses are

(6) $$E\left(U_{i}\right)=1,\;E\left(V_{i}\right)=1,\;E\left(Y_{i}\right)=\mu_{i},$$

respectively, where 1 is a vector of ones. The variances of $V_{i}$ is

(7) $$Var\left(V_{i}\right)=\sigma^{2}J+\left[\begin{array}{cc} \tau_{1}^{2}R_{1} & 0\\ 0 & \tau_{2}^{2}R_{2} \end{array}\right]$$

where $R_{j}$ is the correlation matrix of $V_{ij}$, j=1,2. The variance of $Y_{i}$ is

(8) $$Var\left(Y_{i}\right)=diag\left(\mu_{i}\right)+diag\left(\mu_{i}\right)Var\left(V_{i}\right)diag\left(\mu_{i}\right)$$

where $diag(\mu_{i})$ is the diagonal matrix of $\mu_{i}$. The covariances between the random effects and the responses are

(9) $$Cov\left(U_{i},Y_{i}\right)=\sigma^{2}\mu_{i}^{\prime },\;Cov\left(V_{i},Y_{i}\right)=Var\left(V_{i}\right)diag\left(\mu_{i}\right),$$

respectively.

## 4. Model Estimation

Similar to Ma et al. [10], we adopt an iterative EM-like algorithm for the model estimation. While updating a component in an iteration, which can be either a vector of random effects, regression parameters or dispersion parameters, we keep other unknowns at their current values. Thus, in the subsections below, we treat random effects and/or parameters as known except the ones under discussion.

### 4.1. Prediction of Random Effects

Let the inverse of $Var(Y_{i})$ be denoted by ${Var}^{-1}\left(Y_{i}\right)$, i=1,2,…,m. The values of the random effects are updated by the orthodox BLUPs of the random effects as follows:

(10) $$\hat{U_{i}}=E\left(U_{i}\right)+Cov\left(U_{i},Y_{i}\right){Var}^{-1}\left(Y_{i}\right)\left[Y_{i}-E\left(Y_{i}\right)\right],$$

and

(11) $$\hat{V_{i}}=E\left(V_{i}\right)+Cov\left(V_{i},Y_{i}\right){Var}^{-1}\left(Y_{i}\right)\left[Y_{i}-E\left(Y_{i}\right)\right].$$

where the terms in the equations are given in Equations (6)–(9). As pointed out in [10], the orthodox BLUPs minimize the mean squared distance between the random effects and their predictors within the class of linear functions of the responses. The estimating equations for the regression and random effects parameters can then be constructed based on these predictors.

### 4.2. Estimation of Regression Parameters

As in Ma et al. [5], we may rewrite Equation (1) into a single Poisson mixed model with regression parameters $\gamma ={\left(\alpha^{\prime },\beta^{\prime }\right)}^{\prime }$ as follows:

(12) $$Y_{ijt}|W∼Poisson\left(V_{ijt}\exp\left(x_{ijt}^{\prime }\gamma \right)\right),$$

where $x_{i1t}={\left(z_{it}^{\prime },x_{it}^{\prime }\right)}^{\prime }$ and $x_{i2t}={\left(z_{it}^{\prime },0^{\prime }\right)}^{\prime }$. Thus, we may adapt the orthodox BLUP approach in [10] to our model.

We first differentiate the partially observed “joint” log-likelihood for the data and the random effects with respect to the regression parameters γ to obtain the partially observed “joint” score function. We then have an unbiased estimating equation for γ below, by replacing the random effects with their corresponding orthodox BLUP predictors:

(13) $$\psi \left(\gamma \right)=\sum_{i=1}^{m}\sum_{j=1}^{2}\sum_{t=1}^{T}x_{ijt}^{\prime }\left[y_{ijt}-{\hat{V}}_{ijt}\left(\gamma \right)\mu_{it}\left(\gamma \right)\right]=0.$$

Following Ma et al. [10], Equation (13) can be solved iteratively using the Newton scoring algorithm [15] with γ being updated as follows:

(14) $$\gamma^{*}=\gamma -S^{-1}\left(\gamma \right)\psi \left(\gamma \right)$$

with the explicit expression of the sensitivity matrix given by

$$S\left(\gamma \right)=-\sum_{i=1}^{m}X_{i}^{\prime }diag\left(\mu_{i}\right){Var}^{-1}\left(Y_{i}\right)diag\left(\mu_{i}\right)X_{i}$$

where X is the design matrix formed by stacking $x_{ijt}$’s, i.e., $X_{i}=(x_{i11}$, *…*, $x_{11T}$, $x_{i21}$, *…*, $x_{i2T}{)}^{\prime }$.

The optimality results in Ma et al. [10] still hold, i.e., under mild regularity conditions, the solution to Equation (13) is consistent and asymptotically normal with asymptotic mean γ and asymptotic variance given by the inverse of the negative sensitivity matrix S(γ).

### 4.3. Estimation of Random Effects Parameters

In this subsection, we present a moment approach to estimate the unknown random effects parameters $\sigma^{2}$, $\tau_{j}^{2}$ and $\rho_{j}$, j=1,2.

Following Ma and Jørgensen [10], the dispersion parameter $\sigma^{2}$ for the stock-specific random effects can be estimated in terms of their corresponding orthodox BLUPs ${\hat{U}}_{i}$’s with a bias correction. After some algebraic calculation, the iterative equation for updating $\sigma^{2}$ can be expressed as

(15) $${\hat{\sigma }}_{r}^{2}=\frac{1}{m}\sum_{i=1}^{m}\left\{{\left({\hat{U}}_{i}-1\right)}^{2}+c_{i}\right\},$$

where $c_{i}$ is a bias-correction term defined as

$$c_{i}={\hat{\sigma }}_{r-1}^{2}-{\hat{\sigma }}_{r-1}^{4}\mu_{i}^{\prime }{Var}^{-1}\left(Y_{i}\right)\mu_{i}$$

with ${\hat{\sigma }}_{r-1}^{2}$ as the estimate from the previous iteration. Similarly, the iterative equations for estimating $\tau_{1}^{2}$ and $\tau_{2}^{2}$ are given as

(16) $${\hat{\tau }}_{j,r}^{2}=\frac{1}{mT}\sum_{i=1}^{m}\sum_{t=1}^{T}\left\{{\left({\hat{V}}_{ijt}-{\hat{U}}_{i}\right)}^{2}+d_{ijt}\right\},$$

where the bias-correction term $d_{ijt}$ is expressed as

$$\begin{array}{cc} d_{ijt}= & {\hat{\tau }}_{j,r-1}^{2}-\sigma^{4}\mu_{i}^{\prime }{Var}^{-1}\left(Y_{i}\right)\mu_{i}-Cov\left(V_{ijt},Y_{i}\right){Var}^{-1}\left(Y_{i}\right)Cov\left(Y_{i},V_{ijt}\right)\\ \; & +2Cov\left(U_{i},Y_{i}\right){Var}^{-1}\left(Y_{i}\right)Cov\left(Y_{i},V_{ijt}\right). \end{array}$$

For unstructured correlation structures, $\rho_{j,(t,t^{\prime })}$ can be estimated using an adjusted Pearson estimator as

(17) $$\begin{array}{ccc} {\hat{\rho }}_{j,(t,t^{\prime })} & = & \frac{\sum_{i=1}^{m}\left\{\left({\hat{V}}_{ijt}-{\hat{U}}_{i}\right)\left({\hat{V}}_{ijt^{\prime }}-{\hat{U}}_{i}\right)+b_{ij,(t,t^{\prime })}\right\}}{{\left[\left\{\sum_{i=1}^{m}{\left({\hat{V}}_{ijt}-{\hat{U}}_{i}\right)}^{2}+d_{ijt}\right\}\left\{\sum_{i=1}^{m}{\left({\hat{V}}_{ijt^{\prime }}-{\hat{U}}_{i}\right)}^{2}+d_{ijt^{\prime }}\right\}\right]}^{1/2}}, \end{array}$$

where $b_{ij,(t,t^{\prime })}$ is the correction term which can be simplified as

$$\begin{array}{cc} b_{ij,(t,t^{\prime })}= & \rho_{j,(t,t^{\prime })}\tau_{j}^{2}-Cov\left(V_{ijt},Y_{i}\right){Var}^{-1}\left(Y_{i}\right)Cov\left(Y_{i},V_{ijt^{\prime }}\right)-\sigma^{4}\mu_{i}^{\prime }{Var}^{-1}\left(Y_{i}\right)\mu_{i}\\ \; & +\sigma^{2}\mu_{i}^{\prime }{Var}^{-1}\left(Y_{i}\right)Cov\left(Y_{i},V_{ijt}\right)+\sigma^{2}\mu_{i}^{\prime }{Var}^{-1}\left(Y_{i}\right)Cov\left(Y_{i},V_{ijt^{\prime }}\right) \end{array}$$

For various patterned correlation, we can obtain the patterned correlation matrix from Equation (17). To estimate $\rho_{j}$ under AR(1) structure, it would be sufficient to estimate the lag 1 $\left(\rho_{j}^{1}=\rho_{j}\right)$ correlation only, which can be estimated as

(18) $$\begin{array}{ccc} {\hat{\rho }}_{j} & = & \frac{\sum_{i=1}^{m}\sum_{t=1}^{T-1}\left\{\left({\hat{V}}_{ijt}-{\hat{U}}_{i}\right)\left({\hat{V}}_{ij(t+1)}-{\hat{U}}_{i}\right)+b_{ij,(t,t+1)}\right\}}{{\left[\left\{\sum_{i=1}^{m}\sum_{t=1}^{T-1}{\left({\hat{V}}_{ijt}-{\hat{U}}_{i}\right)}^{2}+d_{ijt}\right\}\left\{\sum_{i=1}^{m}\sum_{t=1}^{T-1}{\left({\hat{V}}_{ij(t+1)}-{\hat{U}}_{i}\right)}^{2}+d_{ij,t+1}\right\}\right]}^{1/2}}. \end{array}$$

### 4.4. Computational Procedures

To start the estimating algorithm, we need some sensible initial values for the unknown parameters. We obtained the initial values ${\hat{\gamma }}_{0}$ of the regression parameters by fitting a Poisson regression to Equation (12) ignoring the random effects. The initial value ${\hat{\rho }}_{j,0}$ of $\rho_{j}$ was taken to be the lag 1 sample correlation of the quarterly counts of daily limit-ups or limit-downs. Similarly, by treating appropriate averages of the counts as rough estimates of the random effects, we obtained the initial values of the dispersion parameters as the sample moments of the random effects. Specifically, the initial value of $\sigma^{2}$ was taken as

$${\hat{\sigma }}_{0}^{2}=\frac{1}{m}\sum_{i=1}^{m}{\left\{\left[\frac{1}{2T}\sum_{j=1}^{2}\sum_{t=1}^{T}Y_{ijt}\right]-1\right\}}^{2}$$

and the initial value of $\tau_{j}^{2}$ was

$${\hat{\tau }}_{j,0}^{2}=\frac{1}{T}\sum_{t=1}^{T}{\left\{\left[\frac{1}{m}\sum_{i=1}^{m}Y_{ijt}\right]-1\right\}}^{2}$$

The algorithm was iterated as follows.

**Step 1:** Initialize the parameters with $\gamma_{0}$, $\sigma_{0}^{2}$, $\tau_{j,0}^{2}$ and $\rho_{j,0}$ as described above.
**Step 2:** At the *r*th iteration,
  (1) Update regression parameters γ by Equation (14),
  (2) Predict the random effects by their orthodox BLUPs given in Equations (10) and (11),
  (3) Update dispersion parameter $\sigma^{2}$ by Equation (15),
  (4) Update dispersion parameter $\tau_{j}^{2}$ by Equation (16),
  (5) Update correlation $\rho_{j}$ by Equation (18).
**Step 3:** Repeat Step 2 until the sum of absolute changes in the parameters is below a prespecified threshold, for example, $10^{-4}$ or $10^{-7}$.

## 5. Analysis of Quarterly Counts of Stock Daily Limit Ups and Limit Downs

We first present some descriptive statistics of the variables in Table 2 and Table 3. From Table 2, the variables PB and PS are highly dispersed; for computational stability consideration, we divided all these three predictors PE, PB and PS by 100 in our analysis. In Table 3, we observe that the counts of limit-ups and limit-downs are positively correlated (r=0.56), and that the counts are also positively correlated with PE and PB although the correlations are weaker.

**Figure 2:**
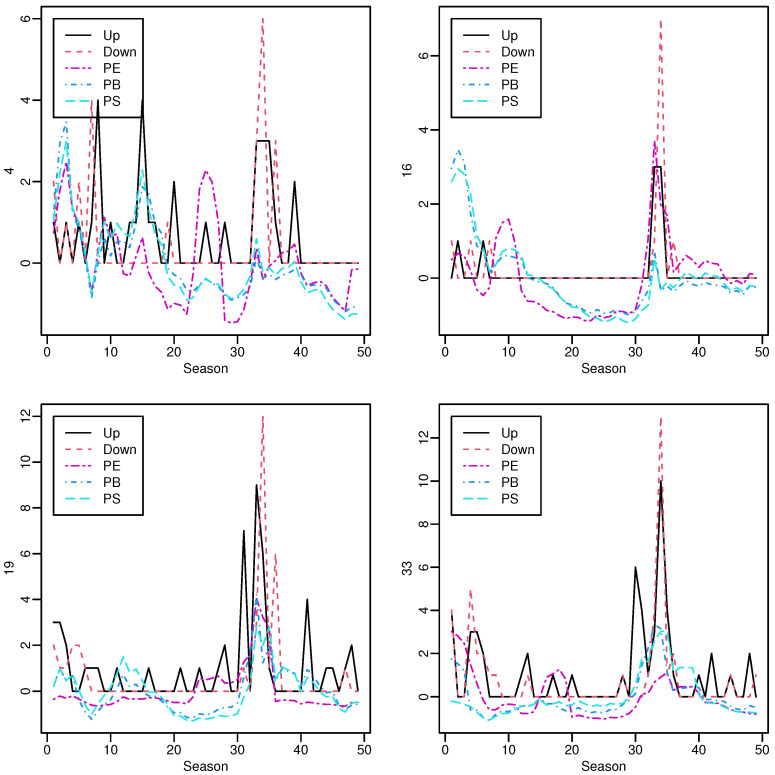
Trace plots of all the variables for four randomly selected stocks. The variables PE, PB and PS were scaled *within the stock*, that is, subtracting the sample mean and then dividing by the sample standard deviation.

The trace plots of all these five variables, limit-ups, limit-downs, PE, PB and PS, are presented in Figure 2 for four stocks, one in each panel. There seems to be some evidence that PE and PB follow the same temporal pattern. Furthermore, PE looks different than PB and PS.

We fitted the proposed model to the stock instability data. The parameter estimation results are presented in Table 4.

The price-to-book ratio variable PB is defined as the stock price divided by the net asset value per share. PB represents the intrinsic value of the stock. The higher the PB is, the lower the intrinsic value of the stock is. The estimated effect of PB on the quarterly count of daily limit-ups was $\beta_{1}^{*}=0.0345$ and significant, with a *p*-value 0.0200<0.05, whereas the estimated effect of PB on the quarterly count of daily limit-downs was $\alpha_{1}=0.0308$ but insignificant, with a *p*-value 0.2070>0.05. Furthermore, the estimated effect of PB on the corresponding binomial proportion was $\beta_{1}=0.0038$, but highly insignificant, with a *p*-value of 0.8938. In other words, only the quarterly count of daily limit-ups was affected by PB significantly, whereas neither the quarterly count of daily limit-downs nor the corresponding binomial proportion was affected by PB significantly.

The price-to-earnings ratio variable PE is defined as the price of a stock divided by the earnings per share. PE represents the valuation of the stock: the higher the PE, the higher the valuation of the stock. The estimated effects of PE on both quarterly counts of daily limit-ups and limit-downs were positive and highly significant. More specifically, the corresponding regression parameters for quarterly numbers of limit-ups and limit-downs were estimated as $\beta_{2}^{*}=9.2711$ and $\alpha_{2}=5.9616$ with *p*-values of 0.0000 and 0.0000, respectively. Furthermore, the estimated effect of PE on binomial proportion was also positive and highly significant with $\beta_{2}=3.3095$ and a *p*-value of 0.0080. That is, with PB and PS being held constant, as PE increased, both quarterly counts of daily limit-ups and limit-downs tended to increase significantly, and so did the corresponding binomial proportion. In other words, as PE increased, the quarterly count of daily limit-ups tended to increase faster than the quarterly count of limit-downs.

The price-to-sales ratio variable PS was defined as the price of a stock divided by the sales per share. None of the estimated effects of PS on the quarterly count of daily limit-ups, the quarterly count of daily limit-downs and the corresponding binomial proportion were significant; these insignificance results of PS were also in agreement with the close-to-zero sample correlations with the counts of limit-ups (r=−0.01) and limit-downs (r=−0.01) in Table 3.

**Figure 3:**
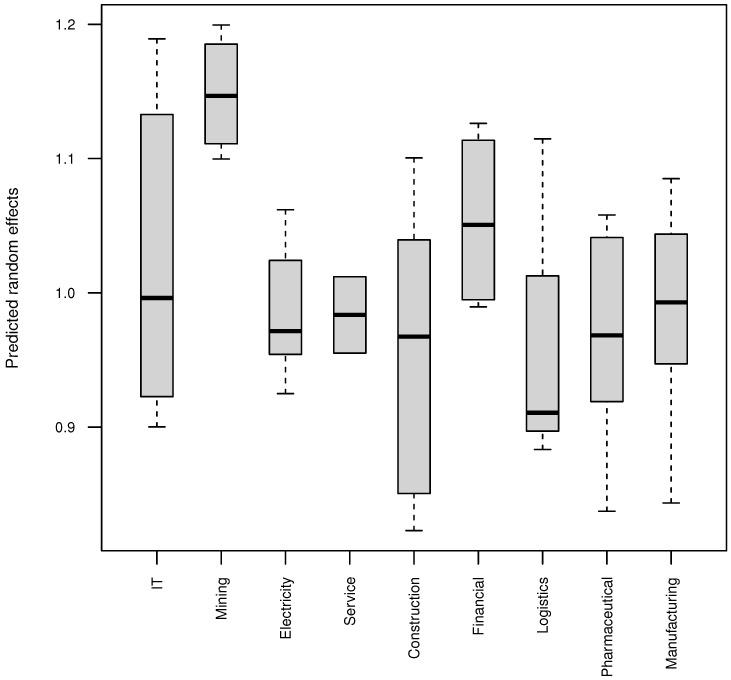
Parallel box plots of the predicted stock-specific random effects by industry.

The stock-specific random effects helped characterize the positive association between quarterly counts of daily limit-ups and limit-downs. The higher the stock-specific random effects, the higher the frequencies of both quarterly counts of daily limit-ups and limit-downs; therefore, the stock-specific random effects reflected stock instabilities. Thus, we term stock-specific random effects as stock-specific instabilities hereafter. We present the parallel box plots of the predicted stock-specific random effects by industry in Figure 3 to assess stock-specific instabilities. First, the mining industry tended to have much higher stock instabilities than other industries. Second, the production and supply of electricity, construction, manufacturing, service and pharmaceutical industries tended to have relatively lower stock instabilities. Third, the IT industry had a wide range of stock instabilities from very low to very high, whereas the financial industry tended to have higher than average stock instabilities with much narrower range. Finally, the logistics industry tended to have very low stock instabilities with an exception.

## 6. Discussion

For longitudinal binomial data, we proposed a joint Poisson mixed model for the number of successes and the number of failures when their association was positive over time. Its usefulness and advantages were demonstrated through the analysis of quarterly data of stock daily limit-ups and limit-downs. Compared with traditional logistic mixed models, the proposed joint model could still assess covariate effects on the binomial proportions through the induced binomial model (see Equation (5)), while the influence of a random number of trials was incorporated. In addition, the ability to allow for zero number of trials was also an advantage as excluding this portion of data might lead to biased inferences on the binomial proportion. Furthermore, the predicted stock-specific random effects enabled the assessment of stock instabilities by industry.

In accordance with the proposed estimation method, we did not specify a parametric form for the random effects distributions. While this formulation enjoys the property of robustness against misspecified random effects, it is in principle straightforward to assume some parametric distributions for the random effects and estimate the parameters using alternative methods. For example, the hierarchical nature of the model makes it easy to implement in Bayesian computing package JAGS or OpenBUGS, which only requires users to specify the model structure.

Finally, the proposed joint Poisson mixed model can be easily extended to longitudinal multinomial data by directly modelling counts of each category with a Poisson mixed model.

## Figures and Tables

**Table 1:** A hypothetical example.

	Time 1	Time 2	Time 3	Time 4	Time 5	Time 6	Time 7	Time 8
Success	10	20	30	40	50	60	70	80
Failure	10	20	30	40	50	60	70	80
Total	20	40	60	80	100	120	140	160

**Table 2:** Summary statistics of the variables.

	Min.	1st Qu.	Median	3rd Qu.	Max.	Mean	SD
Up	0	0	0	1	21	0.76	1.55
Down	0	0	0	0	18	0.51	1.43
PE	−1449.53	17.09	33.29	70.87	3676.97	93.96	271.06
PB	−16.34	1.66	2.73	4.72	100.64	3.94	5.22
PS	0.05	1.17	2.84	6.60	4629.43	13.98	173.92

**Table 3:** Pearson’s sample correlations of the variables.

	Up	Down	PE	PB	PS
Up	1.00	0.56	0.12	0.21	−0.01
Down	0.56	1.00	0.06	0.10	−0.01
PE	0.12	0.06	1.00	0.21	0.17
PB	0.21	0.10	0.21	1.00	0.04
PS	−0.01	−0.01	0.17	0.04	1.00

**Table 4:** Parameter estimates in the model for the analysis of quarterly counts of stock daily limit-ups and limit-downs. (The covariates PB, PE and PS were divided by 100 in the analysis).

	Estimate	Standard Error	*p*-Value
Intercept ($\beta_{0}$)	0.2063	0.0957	0.0311
PB ($\beta_{1}$)	0.0038	0.0283	0.8939
PE ($\beta_{2}$)	3.3095	1.2476	0.0080
PS ($\beta_{3}$)	0.2416	0.4743	0.6106
Intercept ($\beta_{0}^{*}$)	−0.7445	0.0595	0.0000
PB ($\beta_{1}^{*}$)	0.0345	0.0148	0.0200
PE ($\beta_{2}^{*}$)	9.2709	0.6666	0.0000
PS ($\beta_{3}^{*}$)	−0.1230	0.1226	0.3156
Intercept ($\alpha_{0}$)	−0.9508	0.0817	0.0000
PB ($\alpha_{1}$)	0.0308	0.0244	0.2071
PE ($\alpha_{2}$)	5.9614	1.0633	0.0000
PS ($\alpha_{3}$)	−0.3646	0.4606	0.4286
$\sigma^{2}$	0.0295		
$\tau_{1}^{2}$	1.9986		
$\tau_{2}^{2}$	5.4142		
$\rho_{1}$	0.4157		
$\rho_{2}$	0.2880		

## Data Availability

The data are available from http://www.sse.com.cn/ (accessed on 5 January 2020) and http://www.szse.cn/ (accessed on 5 January 2020).
